# β-Hydroxybutyrate Exacerbates Hypoxic Injury by Inhibiting HIF-1α-Dependent Glycolysis in Cardiomyocytes—Adding Fuel to the Fire?

**DOI:** 10.1007/s10557-021-07267-y

**Published:** 2021-10-15

**Authors:** Xiurui Ma, Zhen Dong, Jingyi Liu, Leilei Ma, Xiaolei Sun, Rifeng Gao, Lihong Pan, Jinyan Zhang, Dilan A, Jian An, Kai Hu, Aijun Sun, Junbo Ge

**Affiliations:** 1 Department of Cardiology, Zhongshan Hospital, Human Phenome Institute, Fudan University, Shanghai, 201203 China; 2 Department of Cardiology, Shan Xi Cardiovascular Hospital, Taiyuan, 030024 China; 3 Shanghai Institute of Cardiovascular Diseases, Shanghai, 200032 China; 4 NHC Key Laboratory of Viral Heart Diseases and Key Laboratory of Viral Heart Diseases, Shanghai, China; 5 Academy of Medical Sciences Institutes of Biomedical Sciences, Fudan University, Shanghai, 200032 China; 6 Shanghai Fifth People’s Hospital, Fudan University, Shanghai, 200032 China

**Keywords:** Beta-hydroxybutyrate, Hypoxia, Glycolysis, Hypoxia-inducible factor 1α

## Abstract

**Purpose:**

Ketone body oxidation yields more ATP per mole of consumed oxygen than glucose. However, whether an increased ketone body supply in hypoxic cardiomyocytes and ischemic hearts is protective or not remains elusive. The goal of this study is to determine the effect of β-hydroxybutyrate (β-OHB), the main constituent of ketone bodies, on cardiomyocytes under hypoxic conditions and the effects of ketogenic diet (KD) on cardiac function in a myocardial infarction (MI) mouse model.

**Methods:**

Human peripheral blood collected from patients with acute myocardial infarction and healthy volunteers was used to detect the level of β-OHB. N-terminal proB-type natriuretic peptide (NT-proBNP) levels and left ventricular ejection fractions (LVEFs) were measured to study the relationship between plasma β-OHB and cardiac function. Adult mouse cardiomyocytes and MI mouse models fed a KD were used to research the effect of β-OHB on cardiac damage. qPCR, western blot analysis, and immunofluorescence were used to detect the interaction between β-OHB and glycolysis. Live/dead cell staining and imaging, lactate dehydrogenase, Cell Counting Kit-8 assays, echocardiography, and 2,3,5-triphenyltetrazolium chloride staining were performed to evaluate the cardiomyocyte death, cardiac function, and infarct sizes.

**Results:**

β-OHB level was significantly higher in acute MI patients and MI mice. Treatment with β-OHB exacerbated cardiomyocyte death and decreased glucose absorption and glycolysis under hypoxic conditions. These effects were partially ameliorated by inhibiting hypoxia-inducible factor 1α (HIF-1α) degradation via roxadustat administration in hypoxia-stimulated cardiomyocytes. Furthermore, β-OHB metabolisms were obscured in cardiomyocytes under hypoxic conditions. Additionally, MI mice fed a KD exhibited exacerbated cardiac dysfunction compared with control chow diet (CD)-fed MI mice.

**Conclusion:**

Elevated β-OHB levels may be maladaptive to the heart under hypoxic/ischemic conditions. Administration of roxadustat can partially reverse these harmful effects by stabilizing HIF-1α and inducing a metabolic shift toward glycolysis for energy production.

**Supplementary Information:**

The online version contains supplementary material available at 10.1007/s10557-021-07267-y.

## Introduction

The heart is a high energy-consuming organ; therefore, cardiac energy metabolism is essential for its normal biological and physiological functions [1]. Oxygen plays a critical role in cardiac energy metabolism because mitochondrial oxidative phosphorylation provides 95% of the ATP in the healthy, adult mammalian heart [2–5]. Upon limited oxygen supply, the heart can switch from using oxygen-dependent substrates to more oxygen-efficient energy sources [6]. It has previously been shown that glucose yields 11% more ATP per consumed oxygen atom than fatty acids [7]. However, ketone body oxidation yields more ATP per mole of consumed oxygen than glucose [8]. When the heart experiences hypoxia, such as during a myocardial infarction (MI) or in response to other pathologies, including chronic intermittent hypoxia, sleep apnea, and anemia, the heart can decrease fatty acid oxidation and concomitantly increase its use of glucose and ketone bodies as energy substrates [9]. There is a growing body of evidence showing that the increased utilization of glucose during periods of ischemia is cardioprotective [1, 6, 10]. However, it is not yet clear whether an increased use of ketone bodies under hypoxic conditions is adaptive or maladaptive in the heart.

It has been previously shown that the mammalian heart is capable of avid ketone body uptake and oxidation [11]. Furthermore, the role of ketone bodies in cardiometabolic health has been increasingly recognized. In an ischemia–reperfusion rat model, fasting-induced ketosis [12] and intravenous injection of β-hydroxybutyrate (β-OHB), the main component of ketone bodies [13], have been shown to attenuate ischemic injury. It has also been suggested that the heart is better protected against MI in the fed state with a lower level of ketone bodies compared with the fasted state [14]. However, a recent study has shown that both glucose and β-OHB are positively associated with an increased risk of MI [15]. Dietary carbohydrate intake has been associated with a significant increase in cardiovascular mortality [16] and an increased risk of subsequent coronary artery calcium progression [17]. Furthermore, long-term ketogenic diet-induced β-OHB accumulation has been shown to be detrimental to heart health by promoting cardiac fibrosis [18].

Despite these controversial findings, there is currently limited research on the impact of high levels of ketone bodies on heart metabolic adaptations in response to hypoxia. Here, we aim to investigate whether high ketone body levels can modulate cardiac substrate metabolism and/or induce functional alterations in hypoxic cardiomyocytes and mice after MI surgery.

## Materials and Methods

### Human Peripheral Blood Collection

Human peripheral blood was collected from 45 acute MI patients at Shanxi Cardiovascular Hospital, and peripheral blood samples from 32 healthy volunteers served as controls. The patient characteristics are shown in Supplemental Table S1. The clinical outcomes of the acute MI patients were assessed by N-terminal proB-type natriuretic peptide (NT-proBNP) levels and left ventricular ejection fractions (LVEFs), which were measured by echocardiography 7 days after acute MI. Twenty-six patients without early reperfusion therapy were selected to analyze the relationship between β-OHB levels and cardiac function.

The use of human peripheral blood for scientific purposes was approved by the Ethics Committee of Shanxi Cardiovascular Hospital. All methods were conducted in accordance with the approved guidelines and regulations. Written informed consent was obtained from all patients.

### Adult Cardiomyocyte Isolation and Culture

Cardiomyocytes were isolated from adult, male C57BL/6 mice (6–8 weeks old, weighing 20–22 g) that were obtained from the Shanghai Jiesijie Laboratory Animal Center, according to institutional guidelines. The isolation of adult mouse cardiomyocytes was performed as described previously [19]. After euthanasia, the heart was exposed. Following injection of 7 mL EDTA buffer into the right ventricle, the aorta was clamped with forceps, and the heart was removed. Multiple rounds of injections to the apex of the heart were performed with the following: 10 mL EDTA buffer, 3 mL perfusion buffer, and 30 mL collagenase buffer containing collagen II, collagen IV, and proteins. The heart was then pulled apart using forceps and filtered through a 100-μm strainer to remove large tissue debris.

After three rounds of gravity sedimentation, the adult cardiomyocytes were resuspended in culture media. After enzymatic isolation, the cardiomyocytes were seeded onto laminin-coated coverslips (2 × 10^4^ cardiomyocytes/coverslip) in plating media (M199 culture media [Thermo-Fisher Scientific], penicillin–streptomycin [Gibco], and 5% fetal bovine serum [Gibco]) and cultured at 37 °C for 2 h to facilitate attachment. Then, the cardiomyocytes were cultured in M199 culture media containing 0, 0.1, 0.5, 1, 5, 10, 20, 50, or 100 mM Na-β-OHB (Sigma-Aldrich) (Na-β-OHB media). The cardiomyocytes were cultured in these media for 12 h before hypoxia was induced. The cardiomyocytes used in the roxadustat experiments were cultured in Na-β-OHB media before hypoxia as well as treatment with 50 μM roxadustat (Dalian Meilun Biotechnology). To simulate hypoxia, cardiomyocytes were placed in a hypoxia incubator maintained at 37 °C, 1% O_2_, and 5% CO_2_ for 12 h.

Human AC16 cardiomyocytes were maintained in DMEM (Invitrogen) supplemented with 10% fetal bovine serum (Gibco).

### Animals and Surgical Procedures

Male C57BL/6 mice (6–8 weeks old) were randomly assigned to be fed a normal chow diet (CD; 11 kcal% fat, 20 kcal% protein, 69 kcal% carbohydrates) or a low-carbohydrate, low-protein ketogenic diet (KD; 93.5 kcal% fat, 4.7 kcal% protein, 1.8 kcal% carbohydrates; Dyets #HF93.5) for 4 weeks. After the 4 weeks, permanent ligation of the left anterior descending artery (LAD) was performed to induce MI in the mice. MI surgery was performed as previously described by Erhe Gao et al. [20]. Briefly, mice were anesthetized with isoflurane. A small hole was made at the 4^th^ intercostal space, and with the clamp slightly open, the heart was “popped out” through the hole. MI was induced by placing a 6–0 silk suture slipknot around the left anterior descending coronary artery. After ligation, the heart was immediately placed back into the intra-thoracic space followed by manual evacuation of air and closure of muscle and the skin. Sham-operated mice were subjected to the same surgical procedures without LAD ligation. Echocardiographic analysis was performed 1 day after MI surgery. Mice with LVEFs between 30–45% were used for further experiments and were continued on their respective CD or KD. Echocardiographic analysis was repeated 4 weeks later.

All animal experimental procedures were conducted in accordance with the animal welfare guidelines and utilized the ARRIVE guidelines [21]. All animal protocols were approved by the Animal Care and Use Committee of the Zhongshan Hospital, Fudan University.

### Statistics

All experiments were repeated at least three times. The Student’s *t*-test was used to analyze parametric variables between two groups, and one-way analysis of variance (ANOVA) with a post-hoc test was used to compare parametric variables among three or more groups. Linear regression and Pearson correlation analyses were used to analyze the relationship between β-OHB levels and patient clinical outcomes. All values were presented as the mean ± standard deviation (SD), and *n* was used to refer to the sample size. A *P*-value < 0.05 was considered statistically significant.

### Experimental Setup

Detailed descriptions of the experimental setup and chemicals, including quantitative PCR (qPCR) analysis, western blot analysis, immunofluorescence, ketone body assay, intracellular ATP, live/dead cell staining and imaging, lactate dehydrogenase (LDH) and Cell Counting Kit-8 (CCK-8) assays, echocardiography, 2,3,5-triphenyltetrazolium chloride (TTC) staining, plasmid construction and transfection and RNA interference, *UHPLC-HRMS/MS analysis*, and *Metabolic profiling and pathway analysis*, are given in the Supplementary Methods.

## Results

### β-OHB Levels Were Increased in Response to MI in Humans and Mice

**Fig. 1:**
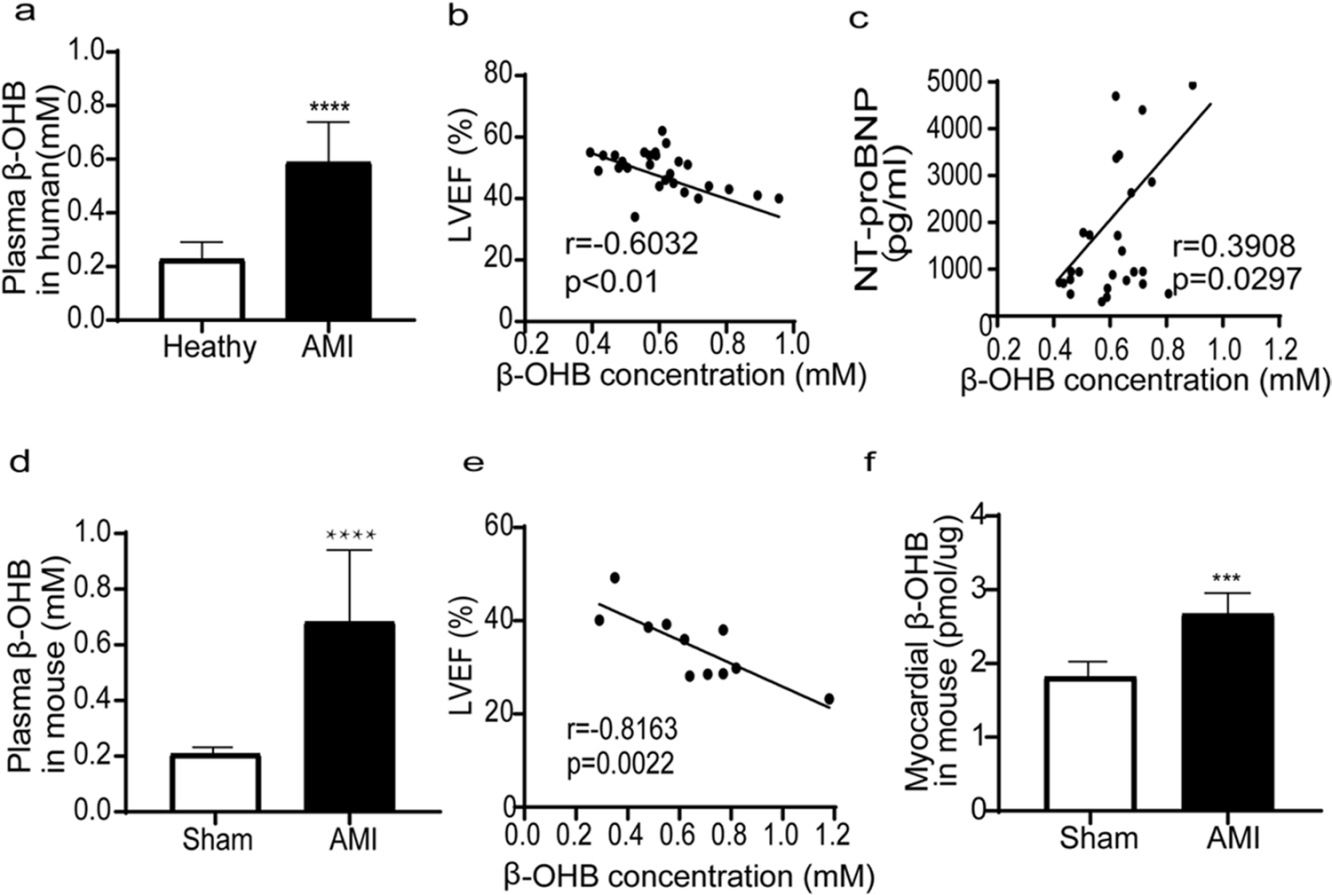
β-OHB levels were increased in response to MI in humans and mice. **a** Plasma β-OHB levels in healthy volunteers (*n* = 32) or AMI patients (*n* = 45), **b** linear regression and Pearson correlation analysis of β-OHB level and LVEF in AMI patients without emergency percutaneous transluminal coronary intervention (*n* = 26), **c** linear regression and Pearson correlation analysis of β-OHB level and NTproBNP in AMI patients without emergency percutaneous transluminal coronary intervention (*n* = 26), **d** mice with similar body weight were randomly assigned to the sham-treated group (*n* = 13) or MI group (*n* = 11), plasma β-OHB level was significantly increased, **e** linear regression and Pearson correlation analysis of plasma β-OHB level and LVEF in mice of MI group (*n* = 11), **f** myocardial β-OHB was significantly increased in mouse 4 weeks after MI (*n* = 11). Data are mean ± SEM. ***P < 0.001, ****P < 0.0001

Baseline characteristics, including age and the proportion of men to women, were not significantly different between the healthy volunteers and acute MI patients (Supplemental Table S1). The serum β-OHB levels were significantly higher in the acute MI patients (Fig. 1a), indicating the in vivo formation of β-OHB during acute MI. The serum β-OHB levels were negatively correlated with LVEFs (Fig. 1b) and were positively correlated with NT-proBNP levels (Fig. 1c) in acute MI patients, indicating that β-OHB may be related to severe myocardial injury.

The serum β-OHB levels were also significantly higher in the MI mice compared with the sham mice (Fig. 1d) and were negatively correlated with LVEFs (Fig. 1e). Additionally, the myocardial β-OHB levels were significantly increased in the mice 3 days after MI surgery (Fig. 1f).

### β-OHB Enhanced Cardiomyocyte Death Under Hypoxic Conditions

**Fig. 2:**
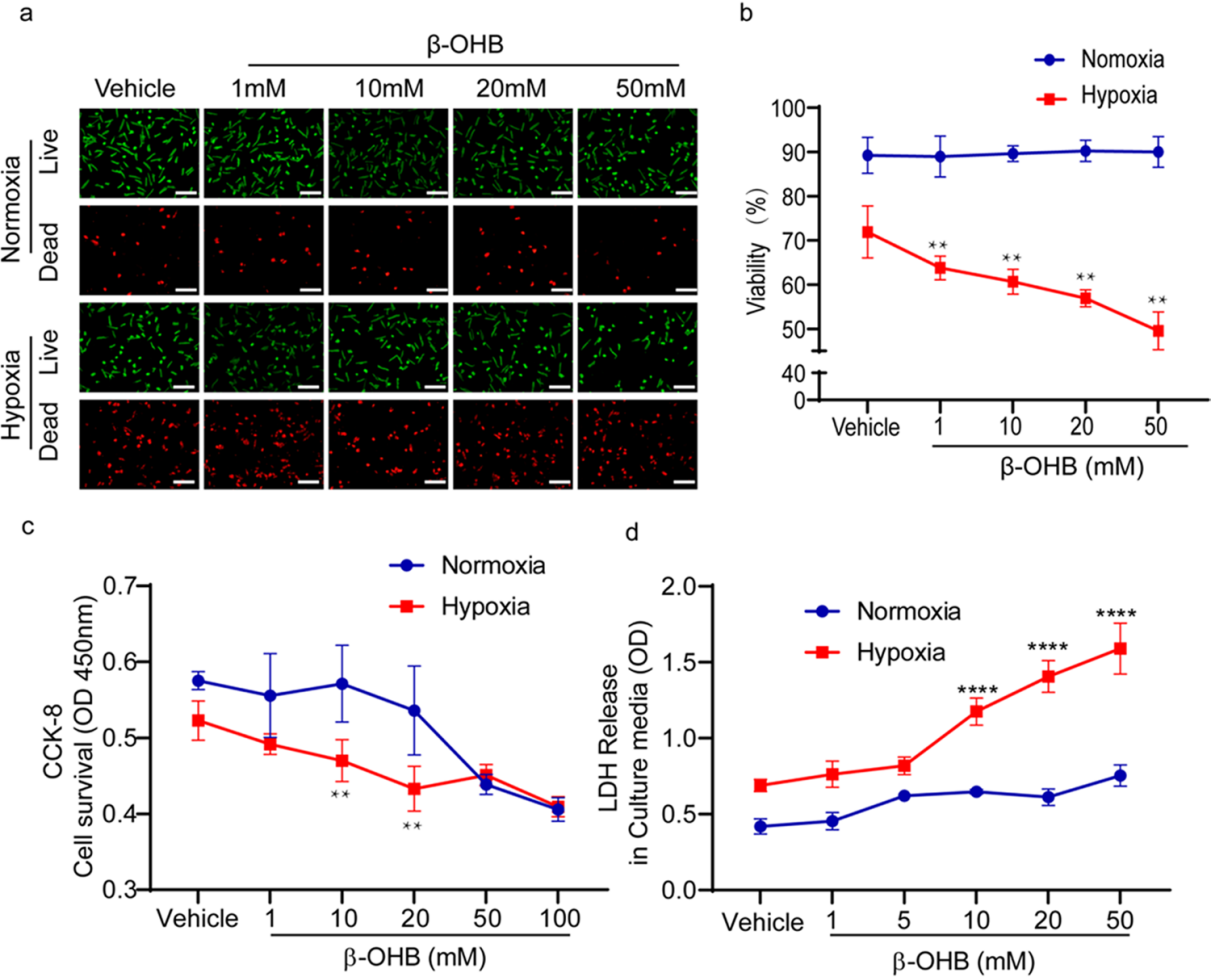
β-OHB enhanced cardiomyocyte death under hypoxic conditions. **a**, **b** Live (green) or dead (red) CMs under normoxia or 12 h of hypoxia in the different dose of β-OHB-treated environment (*n* = 8, different fields), **c** CCK-8 cell survival detection of CMs under normoxia or 12 h of hypoxia in the different dose of β-OHB-treated environment (*n* = 8–12 wells), **d** LDH release in the culture media under normoxia or 12 h of hypoxia in the different dose of β-OHB-treated environment (*n* = 8–12 wells). Data are mean ± SEM. **P < 0.01, ****P < 0.0001 vs. hypoxia 0 h. Scale bars, 50 μm

The live/dead cell assay was used to determine whether β-OHB influenced the death of cardiomyocytes in response to 12 h of hypoxia. Cardiomyocytes were cultured with media containing different doses of β-OHB. Under normoxic conditions, treatment with 1 mM, 10 mM, 20 mM, or 50 mM β-OHB did not affect the viability of cells. However, after 12 h of hypoxia, β-OHB decreased the viability of cells in a dose-dependent manner (Fig. 2a, b). Cell survival was determined using the CCK-8 assay, and results similar to those of the live/dead cell assay were observed (Fig. 2c). As expected, β-OHB treatment increased necrotic cell death, as demonstrated by increased LDH release into the culture media (Fig. 2d). In pilot study, 1–50 mM β-OHB treatment was tested to determine the optimal concentration for the establishment of in vitro model. We found that in the hypoxia state, 10 mM induced significant statistical differences in cell survival, death, and other relevant detection indicators. Therefore, 10 mM β-OHB was selected as the model concentration for this study.

### Effects of β-OHB on Different Metabolites and Metabolic Pathways in Cardiomyocytes Under Hypoxia

**Fig. 3:**
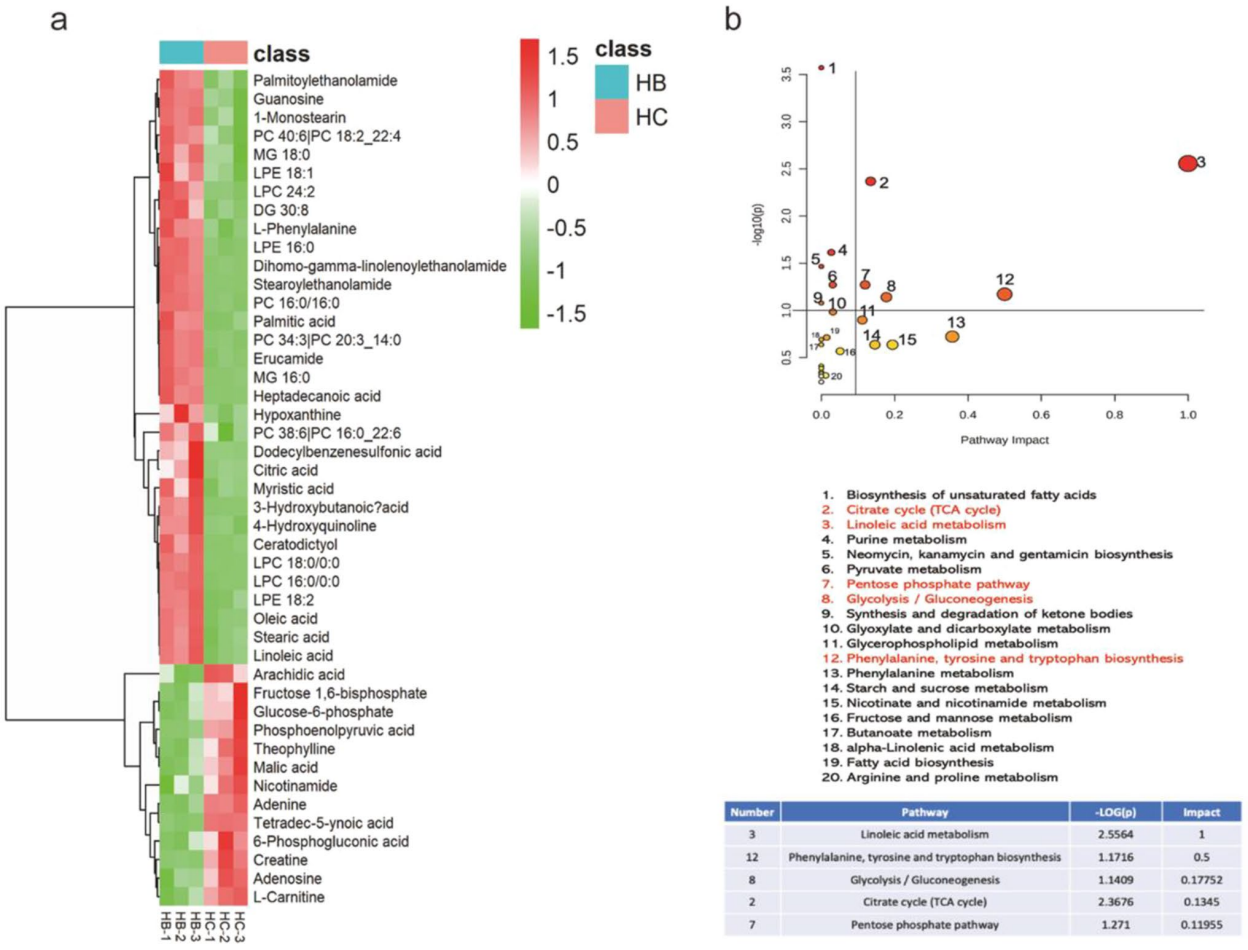
Differential metabolites and metabolic pathways in cardiomyocytes exposed to β-OHB (10 mM) were detected by untargeted metabolomics. **a** Heat map showed the differential metabolites in cardiomyocytes for hypoxia + β-OHB 10 mM (HB) versus hypoxia control (HC) group (*n* = 3 in each group), **b** representative pathway analysis of the metabolites in β-OHB-treated cardiomyocytes under hypoxic condition (HB) versus hypoxia control (HC) group

Through untargeted metabolomics, we found some significantly changed metabolites and involved pathways. Results showed a total of 41 differential metabolites (including positive and negative ion) between hypoxia + β-OHB 10 mM (HB) and hypoxia + control (HC) group, a total of 29 metabolites were increased, and 12 metabolites were decreased in the HB group compared to the HC group (Fig. 3a). To identify the potential hypoxia + β-OHB targeted metabolic pathways, significantly different metabolites were imported into MetaboAnalyst 5.0 (Fig. 3b). According to the criterion of pathway impact > 0.1, *P* < 0.05, 5 metabolism pathways were filtered out to be the most important β-OHB-targeted metabolic pathways under hypoxic condition, which were linoleic acid metabolism, citrate cycle, phenylalanine, tyrosine and tryptophan biosynthesis, glycolysis/gluconeogenesis, and pentose phosphate pathway. β-OHB is an energy substrate, and among the 5 metabolic pathways, we focused on glycolysis affected by β-OHB under hypoxia.

### β-OHB Altered Glycolysis in Cardiomyocytes Under Hypoxic Conditions

**Fig. 4:**
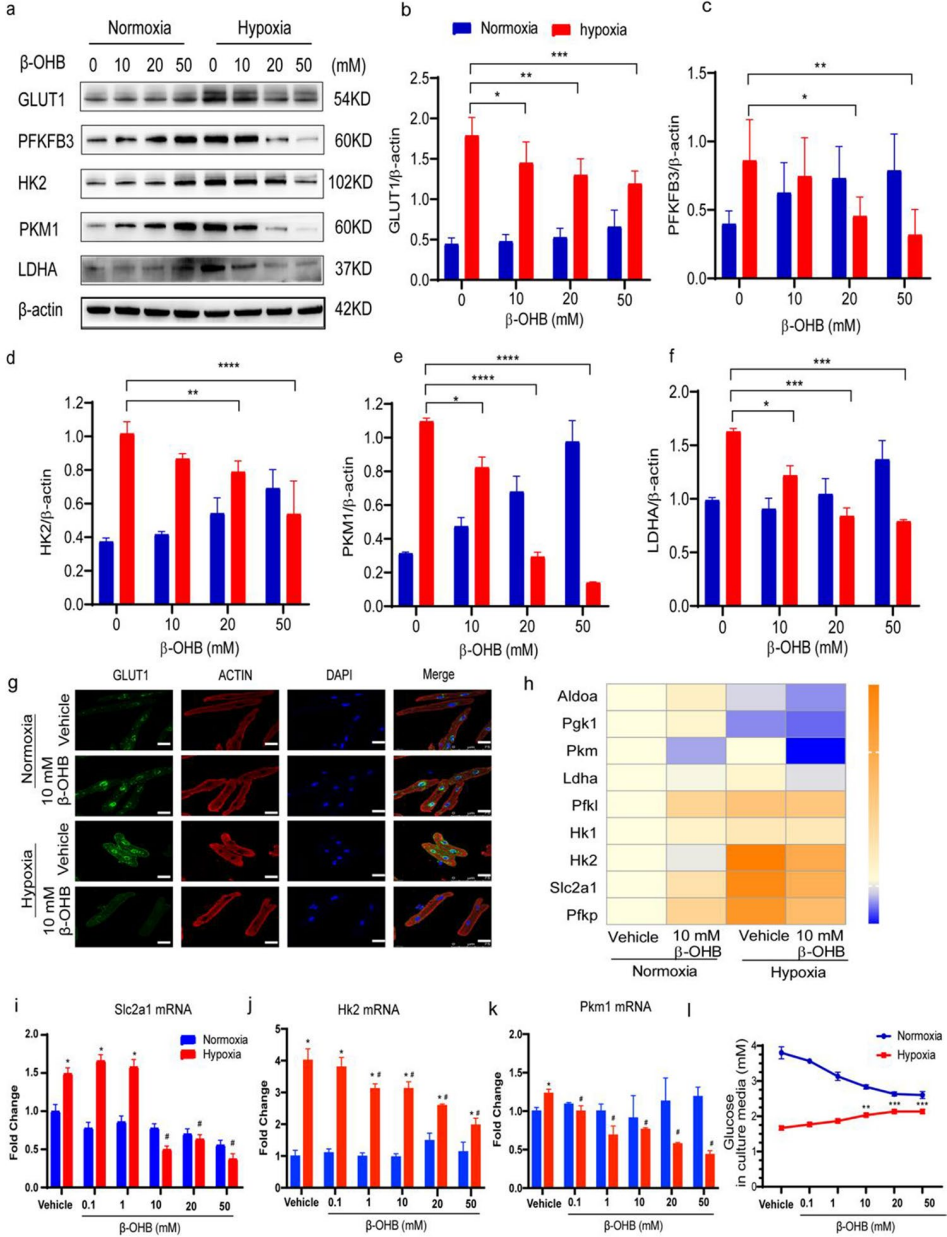
β-OHB altered glycolysis in cardiomyocytes under hypoxic conditions. **a**–**f** Representative western blot of enzymes involved in myocardial glycolysis in CMs cultured with β-OHB at 0 mM, 10 mM, 20 mM, or 50 mM under normoxia or hypoxia. Data are mean ± SEM (*n* = 6 in each group); **g** immunofluorescence imaging showing GLUT1 expression in cardiomyocytes cultured with β-OHB (0 or 10 mM) under normoxia or hypoxia. Scale bar, 25 μm, h qPCR of enzymes involved in myocardial glycolysis in CMs cultured with β-OHB (0 or 10 mM) under normoxia or hypoxia (*n* = 3 in each group); **i**–**k** qPCR of Slc2a1, Hk2, and Pkm1 in CMs cultured with β-OHB (0, 0.1, 1, 10, 20, 50 mM) under normoxia or hypoxia (*n* = 3 in each group), * vs. vehicle in normoxia, ^#^ vs. vehicle in hypoxia; **l** glucose in the culture media which cardiac myocytes cultured with β-OHB at different concentrations under normoxia or hypoxia. Data are mean ± SEM. *P < 0.05, **P < 0.01, ***P < 0.001, ****P < 0.0001, ^#^*P* < 0.05

Our results confirmed that the expression of a glycolytic glucose transporter, GLUT1, was upregulated in cardiomyocytes after 12 h of hypoxia. Accordingly, both the mRNA and protein expression levels of an important glycolytic regulator, PFKFB3, as well as other glycolytic rate-limiting enzymes, including HK2, PKM1, and LDHA, were all upregulated after 12 h of hypoxia compared with the control normoxia group (Fig. 4a–f, h–k).

To further demonstrate the influence of β-OHB on glycolysis in cardiomyocytes, the cells were treated with different doses of β-OHB before undergoing 12 h of hypoxia. Western blot analysis showed that the protein expression levels of GLUT1, HK2, PFKFB3, PKM1, and LDHA decreased in response to β-OHB treatment in a dose-dependent manner compared with the vehicle control group (Fig. 4a–f), suggesting decreased glucose absorption and glycolysis in the β-OHB-treated cardiomyocytes under hypoxic conditions. Furthermore, immunofluorescence revealed low levels of GLUT1 expression in the nuclei and cytoplasm of the β-OHB-treated cardiomyocytes under hypoxia conditions (Fig. 4g). Similar to the western blot results, the mRNA expression levels of the glycolytic enzymes were also decreased in a dose-dependent manner in response to β-OHB treatment under hypoxic conditions (Fig. 4h–k).

Because GLUT1 expression was decreased in response to β-OHB treatment under hypoxia, we measured the glucose concentrations in the culture media as an indicator of cellular glucose uptake. The media glucose concentrations proportionally increased with the dose of β-OHB, indicating that β-OHB may decrease cellular glucose transportation under hypoxia (Fig. 4l). Altogether, these results demonstrated that β-OHB inhibited cellular glucose uptake and glycolysis under hypoxic conditions. Meanwhile, we observed fatty acid oxidation post β-OHB stimulation in hypoxic cardiomyocytes, and the results showed that expression of CPT1B, the rate-controlling enzyme of the long-chain fatty acid beta-oxidation pathway, was not affected by β-OHB in hypoxic cardiomyocytes. ACC, a rate-limiting step in fatty acid biosynthesis, decreased significantly post β-OHB treat cardiomyocytes under hypoxia compared to the hypoxic cardiomyocytes without ß-OHB (Supplementary Figure S1).

### β-OHB Downregulated HIF-1α in Cardiomyocytes in a Dose-Dependent Manner Under Hypoxic Conditions

**Fig. 5:**
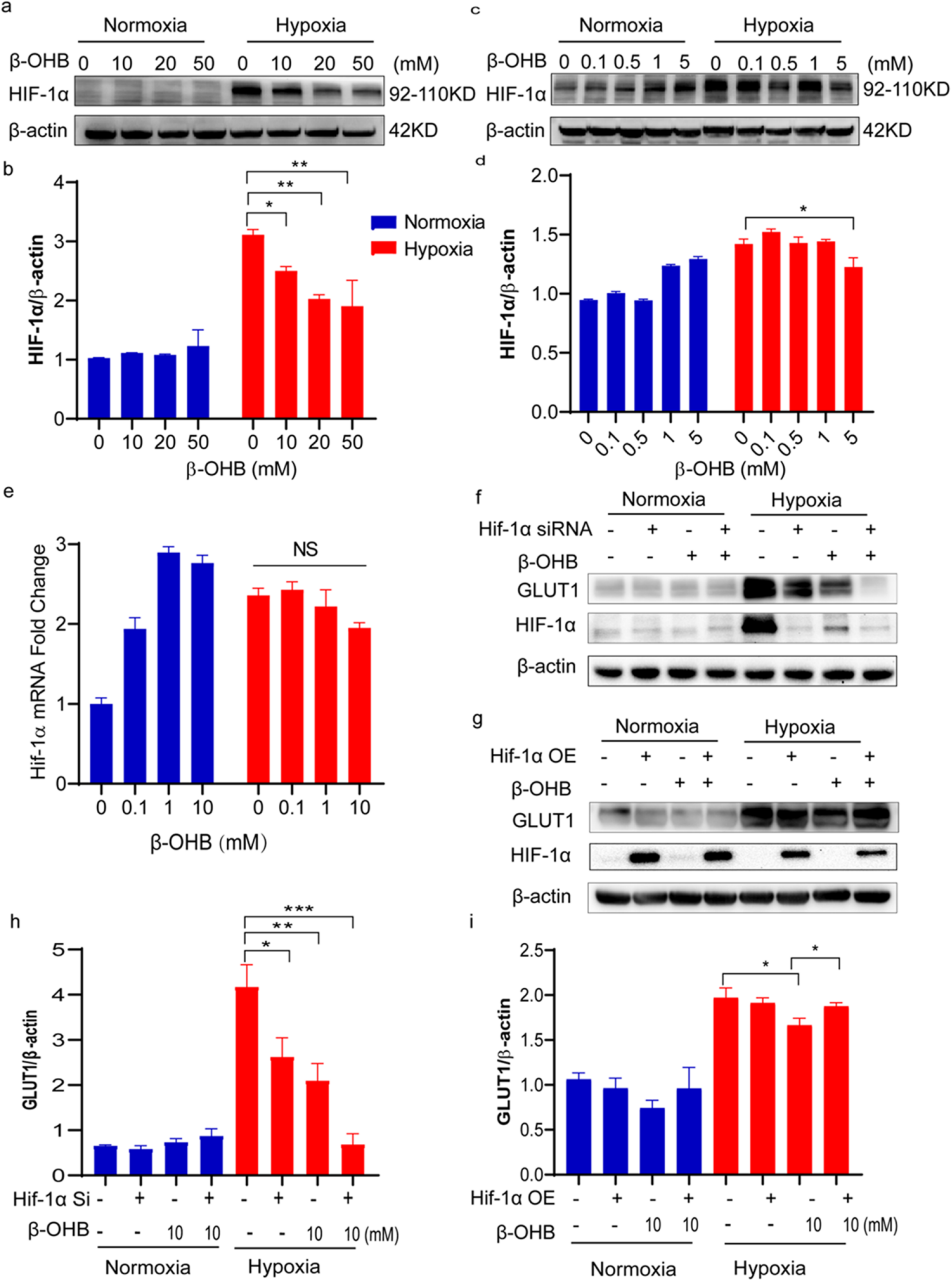
β-OHB downregulated HIF-1α in cardiomyocytes in a dose-dependent manner under hypoxic conditions. **a**–**d** Western blot of HIF-1α in CMs cultured with β-OHB at different concentrations (0, 0.1, 0.5, 1, 5, 10, 20, 50 mM) under normoxia or hypoxia (*n* = 3 in each group), **e** PCR of HIF-1α in CMs cultured with β-OHB (0, 0.1, 1, 10 mM) at different concentrations under normoxia or hypoxia, **f** and **h** western blot of GLUT1 and HIF-1α in HIF-1α siRNA transfection AC16 cells treated with β-OHB 10 mM or not (*n* = 3 in each group), **g** and **i** western blot of GLUT1 and HIF-1α in HIF-1α overexpress (HIF-1α OE) AC16 cells. Data are mean ± SEM. *P < 0.05, **P < 0.01, ***P < 0.001, ****P < 0.0001

**Fig. 6:**
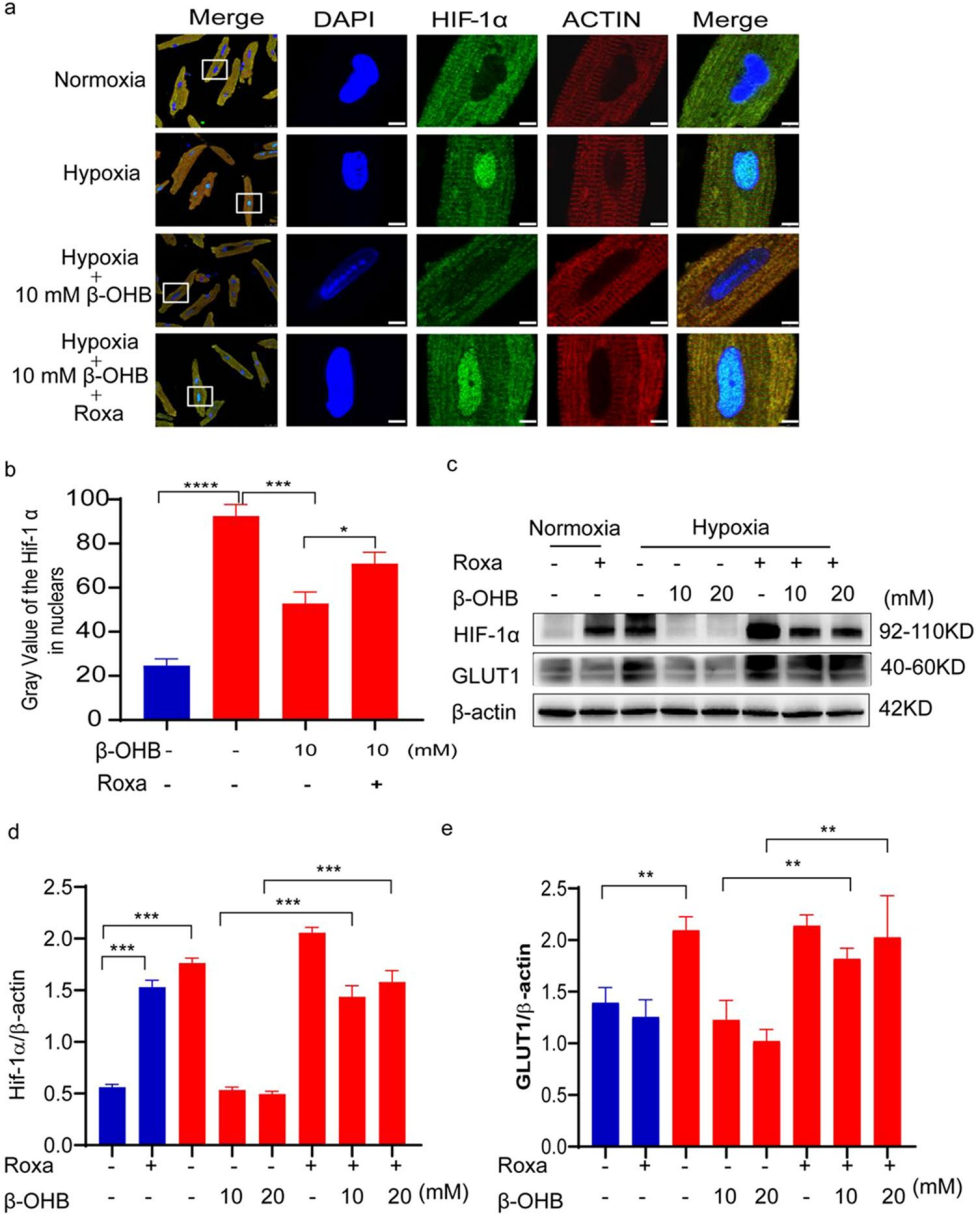

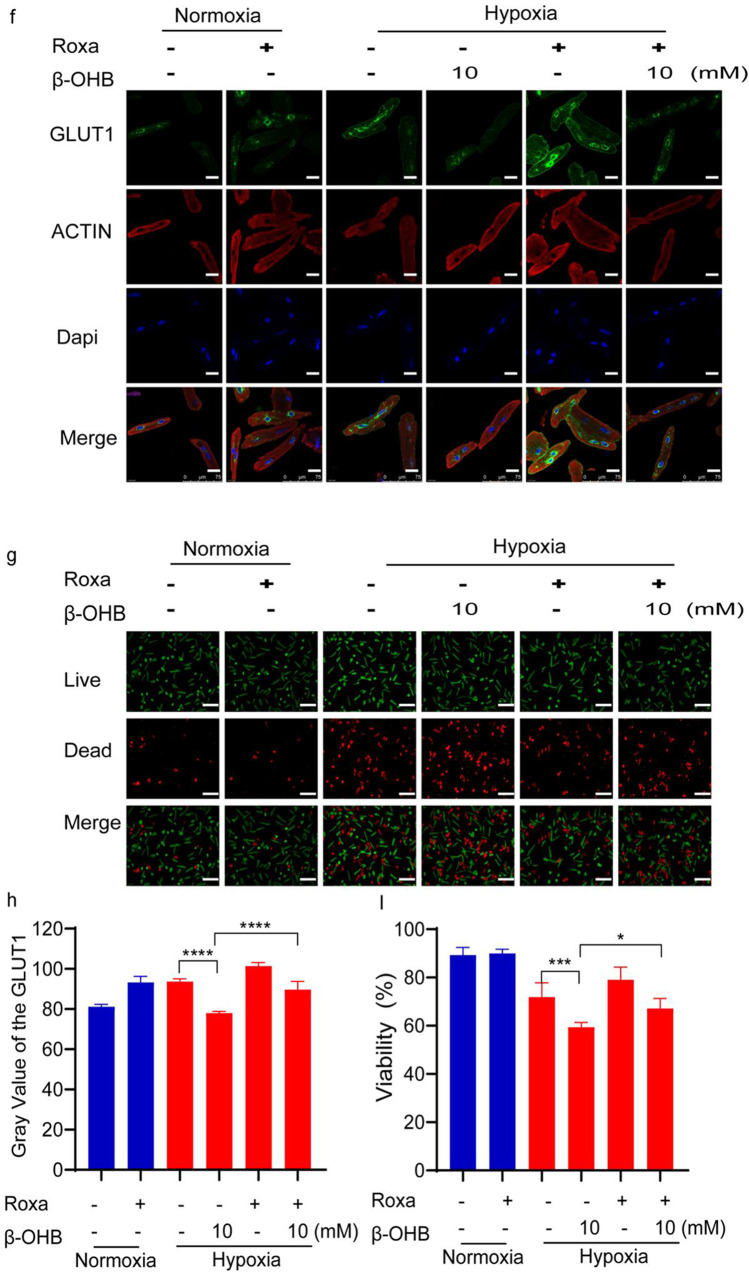
Roxadustat partially reversed the effects of β-OHB in cardiomyocytes under hypoxic conditions. **a** Immunofluorescence imaging showing HIF-1α expression in cardiomyocytes cultured with 50 μM roxadustat and 10 mM β-OHB under normoxia or hypoxia for 12 h, scale bar, 5 μm; **b** quantitation of HIF-1α in the nuclei of CMs cultured with 50 μM roxadustat and 10 mM β-OHB under normoxia or hypoxia for 12 h; **c**, **d**, and **e** western blot of HIF-1α and GLUT1 expression in cardiomyocytes cultured with 50 μM roxadustat and β-OHB (10, 20 mM) under normoxia or hypoxia for 12 h (*n* = 3 in each group); **f** immunofluorescence imaging of GLUT1 in CMs cultured with 50 μM roxadustat and 10 mM β-OHB under normoxia or hypoxia. Scale bars, 25 μm; **g** live (green) or dead (red) CMs cultured with 50 μM roxadustat and 10 mM β-OHB under normoxia or hypoxia, scale bars, 50 μm; **h** quantitation of GLUT1 in CMs cultured with roxadustat and 10 mM β-OHB under normoxia or hypoxia for 12 h (*n* = 3 in each group); **i** quantitation of viability of CMs cultured with roxadustat and 10 mM β-OHB under normoxia or hypoxia. Data are mean ± SEM. *P < 0.05, **P < 0.01, ***P < 0.001, ****P < 0.0001

Next, the expression of glycolysis regulator HIF-1α was analyzed. Consistent with our observation of decreased glycolysis in the β-OHB-treated cardiomyocytes under hypoxia, HIF-1α expression was also significantly downregulated in these cells in hypoxic condition. Cardiomyocytes exhibited significantly higher levels of HIF-1α in response to hypoxia compared with normoxia. However, high-dose β-OHB treatment resulted in decreased HIF-1α expression under hypoxic conditions (Fig. 5a–d). High levels of HIF-1α were detected in the nuclei of cardiomyocytes after 12 h of hypoxia, thus indicating HIF-1α pathway activation. However, these levels were significantly reduced with β-OHB treatment (Fig. 6a, b). HIF-1α mRNA levels were also elevated in the cardiomyocytes in response to hypoxia, but these levels remained unchanged after β-OHB treatment (Fig. 6e), indicating that β-OHB decreased HIF-1α at the posttranslational level.

Additionally, AC16 human cardiomyocyte cells were transfected with HIF-1α siRNA, which resulted in reduced HIF-1α and GLUT1 expression. This result was similar to what was observed in response to β-OHB treatment (Fig. 5f, h). Furthermore, HIF-1α overexpression in AC16 cells suppressed the effects of β-OHB treatment (Fig. 5g, i). These results indicated that β-OHB reduced cellular glucose uptake via the HIF-1α signaling pathway.

### Roxadustat Partially Reversed the Effects of β-OHB in Cardiomyocytes Under Hypoxic Conditions

A HIF prolyl hydroxylase inhibitor, roxadustat (Roxa), was used in further experiments to determine if it could reverse the effects of β-OHB by increasing HIF-1α levels. Western blot and immunofluorescence analyses were used to examine the expression of both HIF-1α and GLUT1 and confirmed that roxadustat administration partially reversed the effects of β-OHB. Immunofluorescence results showed that roxadustat administration partially reversed the effects of β-OHB (Fig. 6a, b). Additionally, western blot confirmed elevated HIF-1α levels after roxadustat administration in cardiomyocytes under normoxic conditions. Roxadustat also resulted in elevated HIF-1α expression in cardiomyocytes under hypoxic conditions after β-OHB treatment (Fig. 6c, d).

Next, GLUT1 expression was evaluated after roxadustat administration as a downstream indicator of HIF-1α activity. Western blot and immunofluorescence analyses both revealed that roxadustat administration increased GLUT1 expression in cardiomyocytes treated with 10 mM β-OHB under hypoxic conditions (Fig. 6c, e, f, h).

Furthermore, the live/dead cell assay showed that roxadustat administration substantially diminished cardiomyocyte death in response to β-OHB treatment under hypoxic conditions (Fig. 6g, i).

### β-OHB Metabolisms Were Obscured Under Hypoxic Conditions in Cardiac Myocytes.

**Fig. 7:**
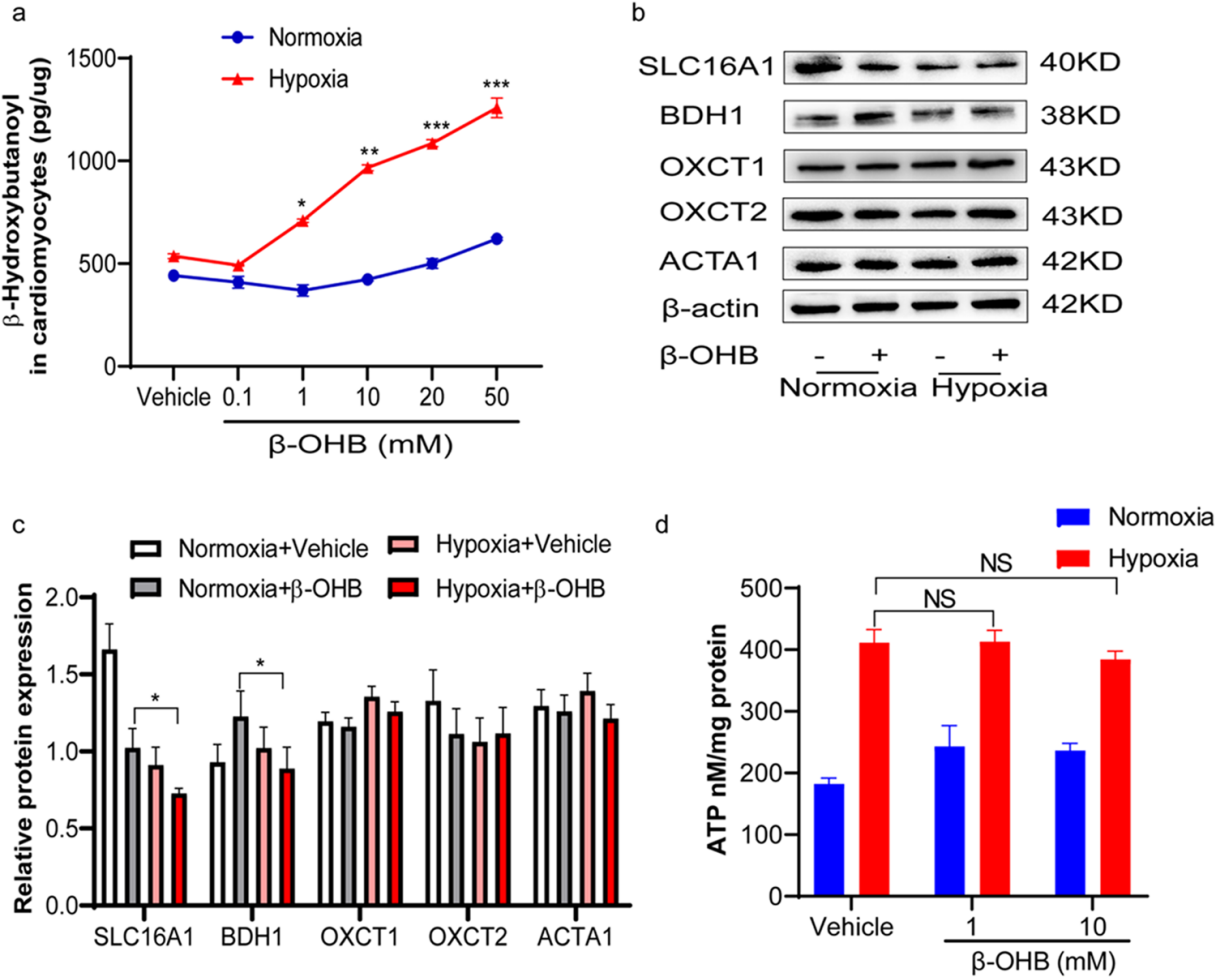
β-OHB metabolisms were obscured under hypoxic conditions in cardiac myocytes. **a** Detected concentration of β-OHB in CMs cultured with β-OHB (0, 0.1, 1, 10, 20, 50 mM) at different concentrations under normoxia or hypoxia; **b** and **c** western blot of SLC16A1, BDH1, OXCT1, OXCT2, and ACTA1 which are β-OHB transporters and the key enzymes for ketone body metabolism in CMs cultured with 10 mM β-OHB under normoxia or hypoxia (*n* = 3 in each group); **d** ATP in CMs cultured with 10 mM β-OHB under normoxia or hypoxia (*n* = 8 in each group). Data are mean ± SEM. *P < 0.05, **P < 0.01, ***P < 0.001

Upon entering the cell, ketone bodies rapidly form acetyl-CoA via a series of reactions catalyzed by BDH1, OXCT1, and mitochondrial acetyl-CoA acetyltransferase 1 (ACAT1). The levels of β-OHB were first detected to observe its metabolism under hypoxic conditions in cardiomyocytes. Under normoxic conditions, β-OHB treatment did not result in elevated β-OHB levels in cardiomyocytes. However, under hypoxic conditions, β-OHB treatment resulted in increased intracellular β-OHB levels in cardiomyocytes in a dose-dependent manner (Fig. 7a). Furthermore, the decreased expression of transporter SLC16A1 and the enzyme BDH1 was observed after β-OHB treatment in hypoxic condition compared with the β-OHB treatment in normoxic condition, suggesting a low ketone body metabolic capacity in the cardiomyocytes under hypoxic conditions (Fig. 7b, c).

Although β-OHB is an energy substrate, there was no change in ATP production in response to β-OHB treatment (Fig. 7d). In contrast to the increased ketone utilization previously reported in end-stage heart failure[22] and HFpEF (heart failure with preserved ejection fraction) [23], the β-OHB treatment did not contribute to cardiomyocyte ATP production under hypoxic conditions, suggesting that the cardiomyocytes did not use ketone bodies as an alternative energy source.

### KD Exacerbated Cardiac Dysfunction in Mice After MI Surgery

**Fig. 8:**
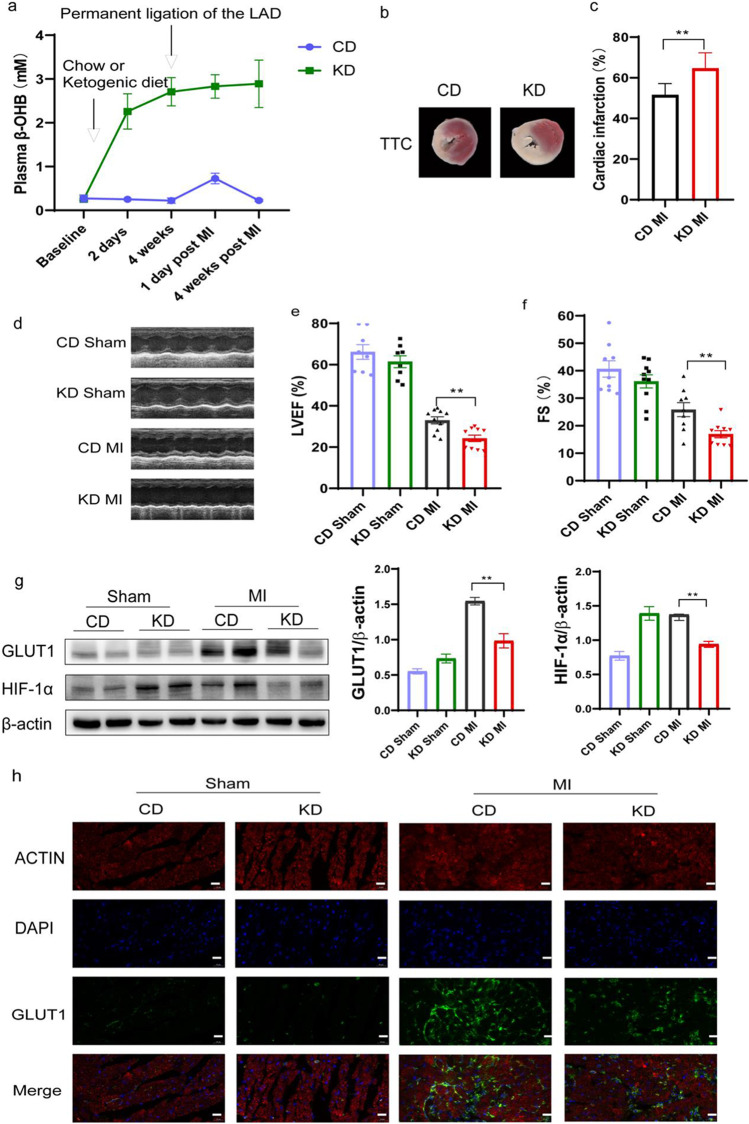
KD exacerbated cardiac dysfunction in mice after MI surgery. **a** The level of β-OHB in plasma of model mice (*n* = 9–11 in each group); **b** triphenyl tetrazolium chloride staining in 4 weeks post-myocardial infarction (*n* = 8 in each group); **c** the infarct area detected by TTC staining was quantified to the left ventricular area; **d**, **e**, and **f** left ventricular ejection fraction (LVEF) and fractional shorting (FS) were measured by echocardiography (*n* = 8–10 in each group); **g** western blot of HIF-1α and GLUT1 analysis from myocardium of KD or CD mice after MI surgery (*n* = 6 in each group); **h** immunofluorescence imaging of GLUT1 in myocardium of KD or CD mice after MI surgery. Scale bars, 20 μm. Data are mean ± SEM. **P < 0.01

Mice were fed either a KD or control CD for 4 weeks prior to MI surgery (Supplemental Table S2). The KD-fed mice exhibited higher plasma β-OHB levels compared with the CD-fed mice (Fig. 8a). Infarct sizes and cardiac function were evaluated 4 weeks after MI surgery. Compared with the CD-fed mice, infarct size was larger in the KD-fed mice (Fig. 8b, c), and cardiac function was worse in the KD-fed mice (Fig. 8d–f) at 4 weeks post MI surgery. These data suggested that KD enhanced MI-induced cardiac injury.

In the KD group, the protein expression of GLUT1 and HIF-1α in the mouse cardiomyocytes post MI was significantly lower than that in the CD group (Fig. 8g). These results suggest that ketone bodies negatively regulate the expression of GLUT1 and HIF-1α. Immunofluorescence further indicated that GLUT1 expression in ischemic area was obviously decreased in KD group than in CD group (Fig. 8h).

## Discussion

**Fig. 9:**
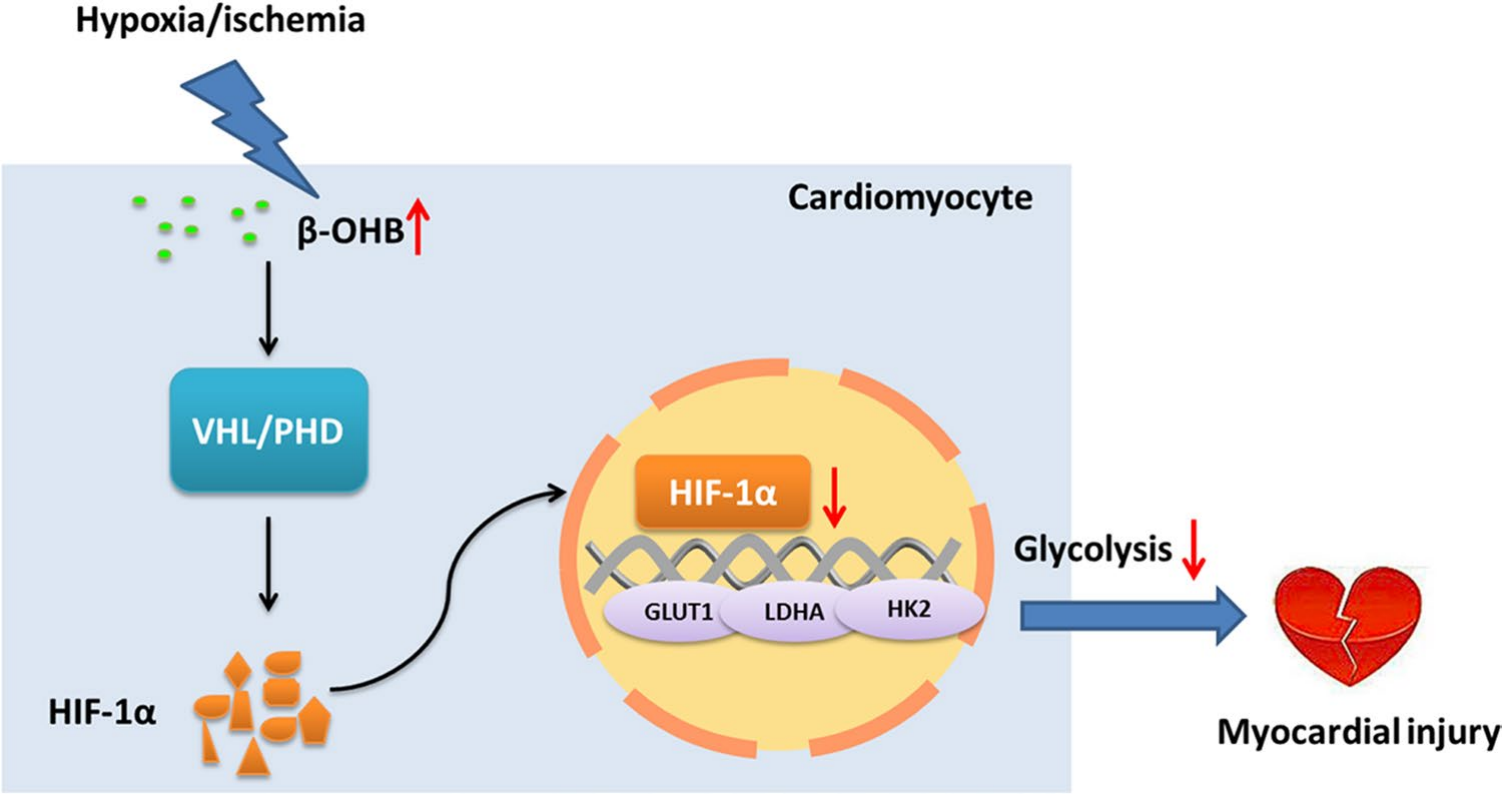
Schematic diagram of how β-OHB exacerbates hypoxic/ischemic myocardial injury. Under hypoxia, β-OHB accumulation occurred in the cardiomyocytes and resulted in HIF-1α destabilization through regulated PHD/VHL. Then β-OHB induced more cardiomyocyte death by decreasing HIF-1α and the downstream GLUT1 and the expression of key glycolytic genes under hypoxic conditions

In this study, we demonstrated that plasma β-OHB levels were elevated in acute MI patients compared with healthy control volunteers, and increased β-OHB levels were also correlated with disease progression. Furthermore, β-OHB treatment resulted in the increased death of adult mouse cardiomyocytes in response to hypoxia as well as larger infarct sizes and deteriorated cardiac function in mice after MI surgery. Metabolic characteristics can influence the function and fate of cardiomyocytes [24]. Under hypoxic conditions, cardiomyocytes have been shown to utilize anaerobic glycolysis instead of oxidative phosphorylation to meet their energy demands and to reduce damage [25]. Here, we showed that β-OHB treatment decreased glycolysis in cardiomyocytes under hypoxic conditions and that downregulated HIF-1α was a key cause of this effect. The HIF prolyl hydroxylase inhibitor, roxadustat, had a therapeutic effect in the β-OHB-treated cardiomyocytes under hypoxic conditions, which was mostly due to increased levels of HIF-1α and GLUT1. In contrast to the alterative ketone utilization observed in advanced-stage heart failure, increased β-OHB utilization was not observed in cardiomyocytes under hypoxic conditions. However, intracellular β-OHB accumulation occurred in these cardiomyocytes and resulted in HIF-1α destabilization (Fig. 9).

Under physiological conditions, ketone bodies are mainly synthesized in the liver and then transported to the whole body for oxidative utilization in the brain, heart, muscle, and other organs [26]. The heart is one of the main ketone body using organs and has weak ketone body synthesis ability [27]. Myocardial infarction is a stress event which may promote ketone bodies synthesis in the liver through neuroendocrine regulation. In our study, the elevated plasma concentration of β-OHB in MI case may mainly come from the liver, but we cannot rule out the possibility that ketone body synthesis is activated in the cardiomyocytes under hypoxic conditions. Song JP’s study showed that elevated β-OHB concentrations have been found to be significantly higher in the hearts of patients with arrhythmogenic cardiomyopathy than in non-diseased donor hearts [27]. Furthermore, increased concentrations of serum ketone bodies, but decreased concentrations of myocardial ketone bodies, have been detected in patients with dilated myocardial disease [28]. In our study, we demonstrated that β-OHB levels were increased in both the serum of acute MI patients as well as the cardiomyocytes of mice after MI surgery; however, β-OHB metabolism was not increased in cardiomyocytes under hypoxic conditions. This observation is obscure because it is contradictory to the alterative ketone utilization observed in advanced-stage heart failure. Previous research has shown that hypertrophied and failing hearts undergo regulatory gene reprogramming to increase the uptake and oxidation of ketone bodies [23, 29]. Specifically, the expression of BDH1 was induced in the hypertrophied and failing mouse heart [22]. However, we found that cardiomyocytes exhibited decreased expression levels of BDH1 in response to hypoxia with β-OHB-treated environment. Studies showed that ketone body metabolism was enhanced and could be a fuel substrate for failing heart. Our study found that under the status of high ketone bodies, hypoxia treatment could not enhance ketone metabolism. The ketone metabolism decreased in hypoxia with high β-OHB environment, suggesting a mismatch between ketone body availability and utilization. In a previous study, it was shown that protein O-GlcNAcylation suppressed BDH1 expression, leading to reduced rates of myocardial β-OHB oxidation [30]. SLC16A1 is proton-dependent transporter and is well characterized due to its critical role in the transport of lactate, pyruvate, and ketone bodies across the plasma membrane. Previous study showed that protein expression of SLC16A1 was not upregulated, while mRNA expression of SLC16A1was significantly decreased post hypoxia because of the lack of corresponding HREs in their promoters [31]. This was consistent with our finding in that in the cardiomyocytes, the expression of SLC16A1 was lower in hypoxia control group compared to normoxia control group. However, the detailed mechanisms of this downregulation are not known. Furthermore, in our study, β-OHB downregulated the protein level of SLC16A1. The expression of SLC16A1 in β-OHB-treated hypoxic cardiomyocytes was decreased more obviously than in the β-OHB-treated normoxic cells. However, Laeger T’s study showed that under conditions of 5.5 mM glucose, 6 mM β-OHB did not alter SLC16A1expression in the hypothalamic GT1-7 neuron cells [32]. Different cell types and β-OHB concentrations may be responsible for the different results. Our data indicate that β-OHB metabolism was obscured and β-OHB was accumulated in the β-OHB-treated cardiomyocytes under hypoxic conditions, so β-OHB itself may participate to modulate the effect in hypoxia.

It is known that enhanced myocardial cell glucose metabolism increases cardiac tolerance to ischemic injury [33]. In this study, glycolysis increased in cardiomyocytes in response to hypoxia; however, elevated levels of β-OHB decreased glycolysis. Therefore, alterations in this metabolic mode due to elevated β-OHB levels may contribute to the decreased adaptability of cardiomyocytes in response to hypoxia. These observations support the hypothesis that ketone body metabolism can regulate the energy source selection of cardiomyocytes under hypoxic conditions.

It is known that HIF-1α regulates the expression of key glycolytic genes, including glucose transporters *GLUT1* and *GLUT4*, *LDH*, phosphoglycerate kinase (*PGK1*), glucose-6-phosphate isomerase (*GPI*), and *PFK1*[34]. Our data showed that β-OHB decreased the expression of HIF-1α in cardiomyocytes under hypoxic conditions and also downregulated the expression of the glycolysis-associated proteins, GLUT1, PKM1, and LDHA. Furthermore, the expression of HIF-1α decreased after β-OHB treatment, and this effect was partially reversed by roxadustat. Therefore, our data collectively indicate that normal glycolysis in cardiomyocytes may be partially regulated by β-OHB via its regulation of HIF-1α under hypoxic conditions. Because HIF-1α is essential for cellular and systemic responses to low oxygen availability, reduced HIF-1α levels may therefore be responsible for the increased death of cardiomyocytes under hypoxic conditions [35]. However, administration of β-OHB resulted in a controversial regulation of the expression of glycolytic enzymes in normoxic and hypoxic conditions. Our results showed that there was no obvious nuclear translocation of HIF-1α in 10 mM β-OHB-treated cardiomyocytes under normoxia (Supplementary Figure S3). So, we concluded that under normoxic conditions, the function of HIF-1α was weak, and regulated GLUT1 expression by β-OHB may be not mainly mediated by HIF-1α in normoxia condition. For β-OHB appear to increase histone acetylation directly through HDAC inhibition [36], our previous study showed that inhibition of HDAC4 can lead to upregulating GLUT1 expression in cardiomyocytes [37], Miyai M’s study showed that increased histone acetylation in cells activated GLUT1 expression [38]. Under normoxia, whether the increased expression of GLUT1 in β-OHB-treated cardiomyocytes is acetylation dependent needs to be further studied. Under hypoxic conditions, cardiomyocytes exhibited high levels of GLUT1 surface expression at the plasma membrane, whereas GLUT1 protein expression was slightly increased under normoxic condition in 10 mM β-OHB-treated cells, and immunofluorescence revealed more GLUT1 perinuclear staining. We suspect that the enriched staining of GLUT1 was largely located in the GLUT-containing vesicles or perinucleus rough endoplasmic reticulum. The subcellular trafficking of GLUT1 between internal vesicular compartments and the cell surface is a major form of GLUT1 regulation [39].

HIF-1 plays a dominant role during cellular adaptation in response to changes in oxygen availability. HIF-1 comprises two subunits: the hypoxia-regulated α subunit, HIF-1α, and the oxygen-insensitive β subunit, HIF-1β [40]. Under normoxic conditions, HIF-1α is rapidly degraded via the von Hippel-Lindau tumor suppressor (pVHL)-mediated ubiquitin–proteasome pathway [41–43]. In our study, β-OHB decreased the protein level of HIF-1α in the cardiomyocytes after hypoxia, but the mRNA levels remained unchanged, and stabilization of HIF by roxadustat through inhibiting the prolyl hydroxylases can partially reverse the effects of β-OHB. However, the mechanisms of β-OHB on HIF-1α signaling might be multiple, and a large part of mechanisms still remains elusive. β-OHB itself can inhibit class I histone deacetylases (HDACs) [36], a family of proteins that play important roles in regulating HIF-1α stability by deacetylating lysine residues [44], and our primary results showed that in cardiomyocytes treated with β-OHB acetylated histone H3 lysine 9 in a dose-dependent manner (Supplement Figure S2a). Furthermore, it is known that ARD1 (arrest defective 1) can mediate acetylation of HIF-1α and negatively regulate HIF-1α stability by accelerating HIF-1α interaction with pVHL under normoxic conditions [45], and our primary results showed that β-OHB can modulate the expression of ARD1 and VHL (Supplementary Figure S2b–c). Next, the metabolism of β-OHB may affect the content of acetyl-CoA, succinate, α-ketoglutaric acid, and pyruvate, which all can modulate the stability of HIF-1α [46–49]. It therefore remains largely unknown which factor will be the principal determinant under hypoxic conditions in cardiomyocytes treated with high β-OHB. Future studies are warranted to clarify related issue.

In the current study, we revealed that the β-OHB/HIF-1α/glycolysis pathway was associated with cardiac injury under hypoxic conditions. However, several questions remain. First, roxadustat only partially reversed the decreased expression of GLUT1 in response to β-OHB treatment under hypoxic conditions, suggesting that it did not completely reverse the effects of the β-OHB-mediated HIF-1α protein degradation. Further studies are required to reveal if β-OHB independently regulates HIF-1α stability. Second, in contrast to what we observed under hypoxic conditions, we observed increased levels of the glycolytic metabolites, PFKFB3, HK2, and PKM1, in β-OHB-treated cardiomyocytes under normoxic conditions. Nonetheless, our goal of this study was to confirm the contribution of the β-OHB/HIF-1α/glycolysis pathway to hypoxic injury. Therefore, we did not investigate the mechanism of these β-OHB-mediated effects under normoxic conditions. Third, besides glycolysis, HIF-1α has many downstream effects. Further studies are required to reveal the regulatory effects of β-OHB on myocardial injury in response to hypoxia. Last, we validated that β-OHB metabolism inhibited HIF-1α-dependent glycolysis in cardiomyocytes under hypoxic conditions. However, the mechanism of this β-OHB-mediated regulation of HIF-1α remains unclear and warrants further investigation.

In conclusion, our results demonstrated that increased β-OHB levels may be maladaptive to cardiomyocytes under hypoxic conditions. Therefore, patients with high levels of β-OHB may experience extensive injury due to ischemic heart disease, and a KD should not be recommended for individuals who have an increased risk of MI.

## Supplementary Information

Below is the link to the electronic supplementary material.

Supplementary file1 (DOCX 1116 KB)

## Data Availability

The data sets generated during and/or analyzed during the current study are available from the corresponding author on reasonable request.
