# Supplementary material for: β-Hydroxybutyrate Exacerbates Hypoxic Injury by Inhibiting HIF-1α-Dependent Glycolysis in Cardiomyocytes—Adding Fuel to the Fire?
Source: Cardiovasc Drugs Ther. 2021 Oct 15;36(3):383–97. doi: 10.1007/s10557-021-07267-y (PMC9090701; doi:10.1007/s10557-021-07267-y)
Supplement: Supplementary file 1 — Supplementary file1 (DOCX 1116 KB) [file 10557_2021_7267_MOESM1_ESM.docx]

***Supplement***

1. ***Supplementary methods***

***1.1 Quantitative PCR (qPCR) analysis***

The cultured cardiomyocytes were collected by centrifugation and lysed in TRIZOL reagent (Invitrogen). RNA was isolated by phase separation and precipitation. RNA yield and quality were assessed by ultraviolet absorbance (NanoDrop ND-1000) and denaturing agarose gel electrophoresis. Complementary DNA was synthetized, and qPCR was performed. Finally, the data were analyzed by the ΔΔCt method.

***1.2 Western blot analysis***

Proteins extracted from the cultured cardiomyocytes were separated by sodium dodecyl sulfate polyacrylamide gel electrophoresis (SDS-PAGE) on a 12% gel and were transferred to a polyvinylidene difluoride (PVDF) membrane (Millipore). After blocking with 5% bovine serum albumin, the blocked membrane was incubated with rabbit polyclonal hypoxia-inducible factor 1α (HIF-1α) antibody (Abcam, ab179483, 1:1,000), rabbit polyclonal voltage-dependent anion channel (VDAC) antibody (Cell Signaling Technology, 4661, 1:1,000), rabbit monoclonal glucose transporter 1 (GLUT1) antibody (Abcam, ab115730, 1:5,000), rabbit monoclonal pyruvate kinase 1 (PKM1) antibody (Cell Signaling Technology, 7067, 1:1,000), rabbit monoclonal 6-phosphofructo-2-kinase/fructose -2,6-biphosphatase 3 (PFKFB3) antibody (Cell Signaling Technology, 13123, 1:1,000), rabbit monoclonal Hexokinase II (HK2) antibody (Cell Signaling Technology, 2867, 1:1,000), rabbit polyclonal lactate dehydrogenase A (LDHA) antibody (Cell Signaling Technology, 2012, 1:1,000) rabbit monoclonal β-hydroxybutyrate dehydrogenase 1 (BDH1) antibody (Abcam, ab193156, 1:1,000), 3-oxoacid CoA-transferase 1 (OXCT1) antibody (Abcam, ab105320, 1:2,000), oxoacid CoA-transferase 2 (OXCT2) antibody (Abclonal, a14920, 1:1,000), solute carrier family 16 member 1 (SLC16A1) antibody (Sigma, HPA003324, 1:1,000), rabbit monoclonal prolyl hydroxylase-2 (PHD2) antibody (Cell Signaling Technology, 4835, 1:1,000), rabbit polyclonal to Von Hippel Lindau (VHL) antibody (Abcam, ab83307, 1:1,000) and rabbit monoclonal to Actin (β-actin) antibody (Abcam, ab179467, 1:5,000). Then, an enhanced chemiluminescence detection reagent (Thermo Scientific™ SuperSignal™ West Pico PLUS, Cat# 34577) was used for imaging.

***1.3 Immunofluorescence***

Sections were incubated with rabbit anti-mouse polyclonal HIF-1α antibody or rabbit anti-mouse monoclonal GLUT1 antibody. An Alexa Fluor 549 donkey anti-rabbit secondary antibody (Thermo-Fisher Scientific) was used for fluorescence imaging. After counterstaining with DAPI, the sections were photographed with a fluorescence confocal microscope (Nikon TE2000).

***1.4 Ketone body assay***

The β-OHB levels in cultured cardiomyocytes were determined using the High Sensitivity β-Hydroxybutyrate Assay Kit (Sigma-Aldrich, MAK272), according to the manufacturer’s directions. The assay was performed in triplicate, and the supplied standard was used for comparisons. Prior to addition to the reaction, samples were deproteinized by filtration using a 10-kDa molecular weight cut-off (MWCO) spin filter. Next, 25 µL of each sample was added to the wells of a 96-well plate. Samples were brought to a final volume of 50 µL with the β-OHB Assay Buffer. Then, 50 μL of the Master Reaction Mix was added to each of the wells containing the samples or standard controls. The plate was incubated for 30 min at room temperature in the dark, and then the fluorescence intensity was measured (λ_ex_=535/λ_em_=587 nm). We calculated β-OHB concentrations according to the standard curve. Ketone concentrations in plasma samples were normalized by volume whereas the protein concentration was measured by bicinchoninic acid assay (BCA) to determine the protein-corrected ketone concentrations in myocardium.

***1.5 Intracellular ATP***

Intracellular ATP was determined using the Enhanced ATP Assay Kit (Beyotime, S0027), according to the manufacturer’s protocol.

***1.6 Live/dead cell staining and imaging***

Staining of the cultured cardiomyocytes was performed using the LIVE/DEAD Viability/Cytotoxicity Kit for mammalian cells (Thermo Scientific), according to the manufacturer’s instructions. Briefly, Hoechst 33342 (Thermo Scientific), calcein, and ethidium dyes were diluted to the final concentrations of 10 μg/mL, 1 μmol/L, and 0.5 μmol/L, respectively, in culture medium made using phenol red-free M199 (Thermo Scientific). The cardiomyocytes were incubated in this medium for 30 min at room temperature and were then washed gently with fresh phenol red-free culture medium and imaged using a standard fluorescence microscope.

***1.7 Lactate dehydrogenase (LDH) and Cell Counting Kit-8 (CCK-8) assays***

LDH assay kits (Beyotime Biotechnology) were used to measure LDH released into the culture medium by cardiomyocytes. The CCK-8 assay (Beyotime Biotechnology) was used to detect cell viability.

***1.8 Echocardiography***

Mice were anesthetized with 2% isoflurane and were then subjected to transthoracic echocardiography using a VeVo 2100 Imaging System (VisualSonics) to assess cardiac structure and function at 1 day as well as 4 weeks after MI surgery. Body temperature was maintained between 36.9–37.3°C, and the heart rate was maintained between 400–500 bpm. Echocardiographic M-mode tracings were recorded, and echocardiographic parameters, including LVEF, fractional shortening (FS), left ventricular end systolic diameter (LVESD), left ventricular end diastolic diameter (LVEDD), and stroke volume (SV), were calculated.

***1.9 2,3,5-triphenyltetrazolium chloride (TTC) staining***

At both 24 h and 3 days after MI surgery, the hearts of the mice were harvested and cut into 1-mm slices. The slices were then incubated with 1% w/v TTC at 37°C for 15 min, followed by fixation with 10% formalin for 20 min. The area not stained by TTC was defined as the infarcted area (white area). Slices were photographed and analyzed by Image Pro Plus 6.0 (National Institutes of Health, Bethesda, MD, USA) to quantify infarcted area and left ventricle size.

***1.10 Plasmid construction and transfection***

HIF-1α was cloned into the GTP-C-3Flag-H vector containing a C-terminal Flag tag. The primers and restriction enzyme sites used for the plasmid construction are listed in Table S3. Lipofectamine 3000 (Invitrogen) was used for plasmid transfections, according to the manufacturer’s instructions. DMEM (500 μL) was added to two clean Eppendorf tubes, and 3 μL of Lipofectamine 3000 was added to one of the tubes and mixed for 5 min. Plasmid was added to the other tube, and its contents were then added to the medium containing Lipofectamine 3000, mixed, and allowed to stand for 20 min before being added to the cardiomyocyte culture medium for transfection. The cell culture medium was replaced with serum-free medium because serum interferes with the Lipofectamine 3000 transfection efficiency. After 6 h incubation with the transfection reagents, the medium was replaced with fresh, normal medium. After 24 h, the cardiomyocytes were subjected to hypoxia for 12 h and then collected for further analyses.

***1.11 RNA interference***

Small interfering RNA (siRNA) was used to achieve stable knockdown in this study. Briefly, double-stranded siRNA targeting HIF-1α was purchased from ZORIN and transfected into cells using RNAiMax (Invitrogen), according to the manufacturer’s instructions. The siRNA sequences are listed in Supplemental Table S3. The knockdown efficiency was verified by qPCR and Western blot.

***1.12 UHPLC-HRMS/MS analysis***

UHPLC-HRMS/MS analysis was performed on ThermoFisher Ultimate 3000 UHPLC and ThermoFisher Q Exactive™ Hybrid Quadrupole-Orbitrap™ Mass Spectrometry (QE). Chromatographic separation was performed on a ThermoFisher Ultimate 3000 UHPLC system with a Waters ACQUITY UPLC BEH C18 column (2.1mm × 100 mm, 1.7μm).

***1.13*** ***Metabolic profiling and pathway analysis***

The raw data of UHPLC-HRMS/MS were firstly transformed to mzXML format by ProteoWizard and then processed by XCMS and CAMERA packages in R software platform. For multivariate statistical analysis, the normalized data were preprocessed by par scaling and mean centering before performing PCA, PLS-DA, and OPLS-DA. For univariate statistical analysis, the normalized data were analyzed in the “muma” software package in R platform, where parametric test was performed on the data of normal distribution by Welch’s t test, while nonparametric test was performed on the data of abnormal distribution by Wilcoxon Mann-Whitney test. Differential metabolites were analyzed and screened according to Volcano Plot. The p values of univariate statistical analysis lower than 0.05 and fold change (FC) larger than 1.2 were identified as potential differential metabolites. Metabolic pathway analysis was performed with KEGG (Kyoto Encyclopedia of Genes and Genomes http://www.kegg.jp/), MetaboAnalyst 5.0 (http://www.metaboanalyst. ca/MetaboAnalyst/) and HMDB (The Human metabolome database http://www.hmdb.ca/).

1. ***Supplementary Figures***

S1

***
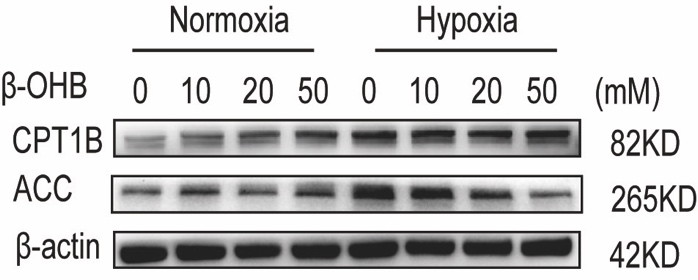
***

Figure S1: Western blot of CPT1B and ACC in CMs cultured with β-OHB at 0 mM, 10 mM, 20 mM, or 50 mM under normoxia or hypoxia. CPT1B, carnitine palmitoyl transferase 1B; ACC, Acetyl-CoA carboxylase.

S2


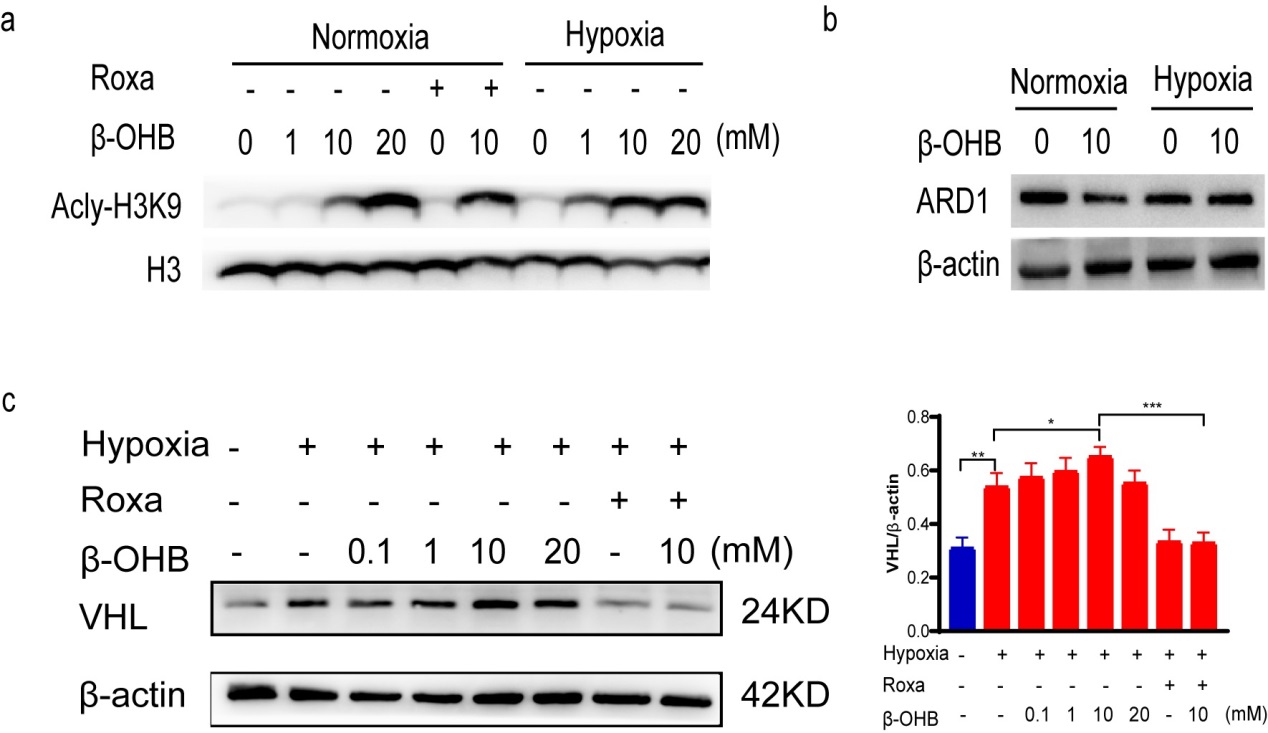


Figure S2: **a** Western blot of Acly-H3K9 and H3 in CMs cultured with β-OHB at 0 mM, 10 mM, 20 mM with or without roxadustat under normoxia or hypoxia. **b** Western blot of ARD1 in CMs cultured with β-OHB at 0 mM or 10 mM under normoxia or hypoxia. **c**. Western blot of VHL in CMs cultured with β-OHB at 0 mM, 0.1 mM, 1 mM, 10 mM, 20 mM with or without roxadustat under normoxia or hypoxia. Data are mean±SEM. *P<0.05, **P<0.01, ***P<0.001.

S3


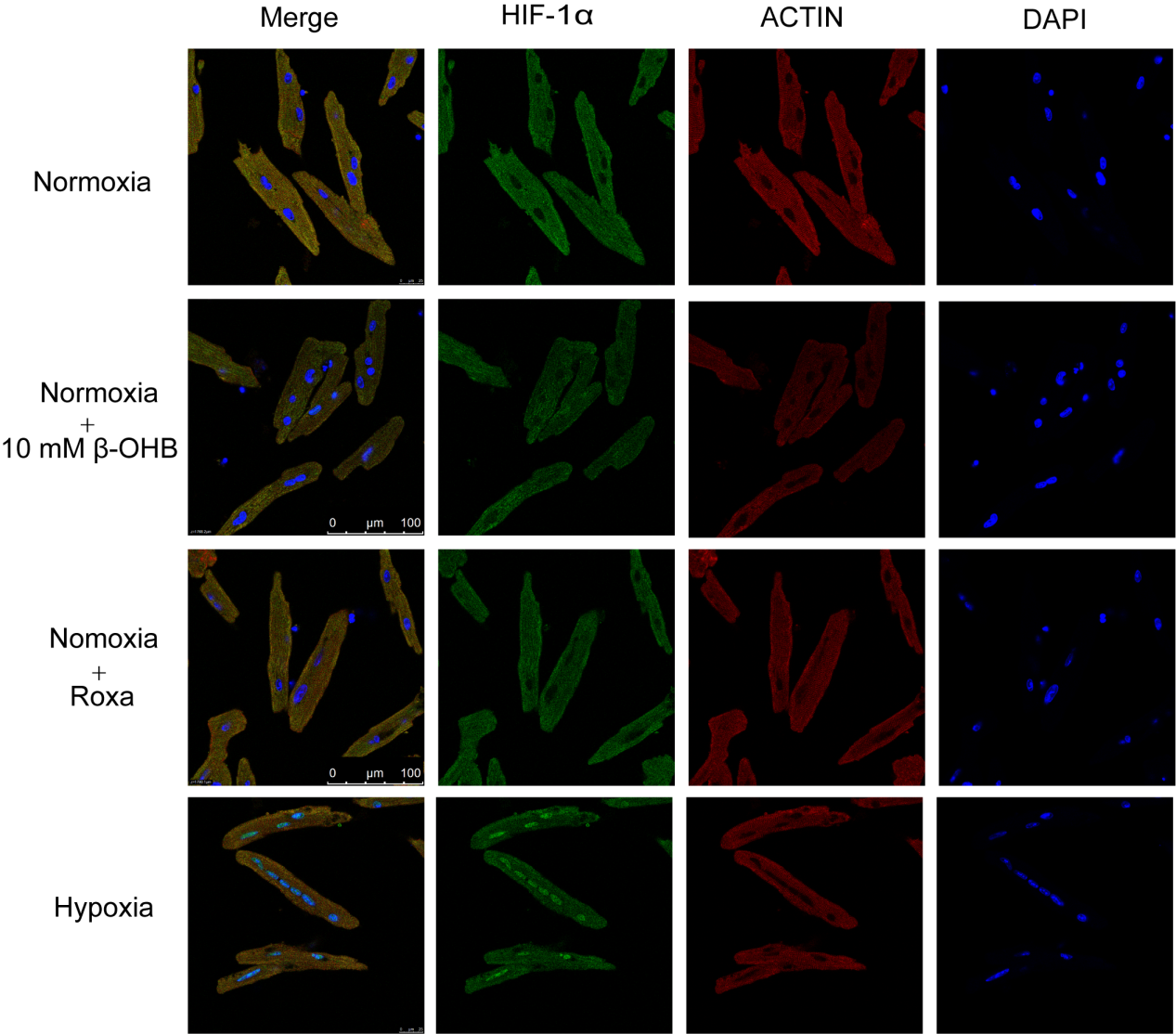


Figure S3: Immunofluorescence imaging showing HIF-1α expression in cardiomyocytes cultured with 10 mM β-OHB or roxadustat under normoxia for 12 h.

S4


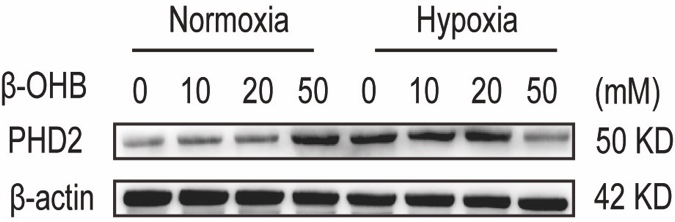


Figure S4: Western blot of PHD2 in CMs cultured with β-OHB at 0 mM, 10 mM, 20 mM, or 50 mM under normoxia or hypoxia.

1. ***Supplementary Tables***

***3.1 Baseline characteristics between healthy volunteers and patients with AMI***

|  | Healthy volunteers | AMI | P |
| --- | --- | --- | --- |
| N | 32 | 45 | - |
| Male, n (%) | 32 (100%) | 45 (100%) | 1 |
| Age, years | 57.25±7.06 | 61.60±11.84 | 0.0627 |
| Diabetes diagnosis, % | 0 | 0 | 1 |
| Hypertension diagnosis, % | - | 16 (35.6%) | - |
| Emergency percutaneous transluminal coronary intervention, % | - | 28 (62.2%) | - |
| In-hospital mortality | - | 2 (4.4%) | - |

***3.2 Body weight, blood β-OHB and blood glucose determined after 4 weeks of diet (n=12 in each group).***

|  | CD | KD |
| --- | --- | --- |
| Body weight | 21.88±0.14 | 17.88±2.47* |
| Blood β-OHB (mM) | 0.27±0.08 | 2.71±1.12* |
| Blood glucose (mM) | 7.57±1.04 | 5.34±1.66* |
| Blood total cholesterol (mMol/L) | 2.32±0.86 | 3.69±1.09* |
| Blood triglyceride (mMol/L) | 1.23±0.35 | 1.04±0.24 |
| LDL-C (mmol/L) | 2.11±0.39 | 3.94±1.21* |
| ALT | 56.78±7.01 | 71.56±17.01* |

***3.3 Sequences of primers used for siRNA and qPCR analyses used in this study.***

| HIF-1α (human) siRNA-1 sense | GGAAAUGAGAGAAAUGCUUTT |
| --- | --- |
| HIF-1α (human) siRNA-1 antisense | AAGCAUUUCUCUCAUUUCCTC |
| HIF-1α (human) siRNA-2 sense | GCUGGAGACACAAUCAUAUTT |
| HIF-1α (human) siRNA-2 antisense | AUAUGAUUGUGUCUCCAGCGG |
| HIF-1α (human) siRNA-3 sense | CCGGUUGAAUCUUCAGAUATT |
| HIF-1α (human) siRNA-3 antisense | UAUCUGAAGAUUCAACCGGTT |
| HIF-1α (human) Plasmids Construction forward | TAGAGCTAGCGAATTCATGGAGGGCGCCGGCGGC |
| HIF-1α (human) Plasmids Construction forward | CTTTGTAGTCGGATCCGTTAACTTGATCCAAAGCTCTGAG |
| Aldoa Forward | AGAAGGTCCTGGCGGCTGTC |
| Aldoa Reverse | TGTGCGACGAAGTGCTGTGAC |
| Pgk1 Forward | AATGGAGCCAAGTCCGTTGTCC |
| Pgk1 Reverse | TGGCACAGGCATTCTCGACTTC |
| Pkm Forward | TGGTGACGGAGGTGGAGAATGG |
| Pkm Reverse | GTCGGCTGCCTTGCGGATG |
| Pfkl Forward | GCATCAAGCAGTCAGCCTCAGG |
| Pfkl Reverse | AGCCAGGTAGCCACAGTAGCC |
| HK-1 Forward | CCTCCGTCAAGATGCTGCCAAC |
| HK-1 Reverse | CCGCCGAGATCCAGTGCAATG |
| Hk2 Forward | GTGTGGAACTGGTGGACGGAGA |
| Hk2 Reverse | GTCATCCAGGCAGCCGTTGTC |
| Slc2a1 Forward | GCAGTTCGGCTATAACACTGG |
| Slc2a1 Reverse | GCGGTGGTTCCATGTTTGATTG |
| Hif-1a Forward | CTGCCACTGCCACCACAACTG |
| Hif-1a Reverse | TGCCACTGTATGCTGATGCCTTAG |
| Pfkp Forward | CAGAGCCACCAGAGGACCTTCG |
| Pfkp Reverse | CAGTCGGCACCGCAAGTCAAG |
| beta-actin Forward | GTGACGTTGACATCCGTAAAGA |
| beta-actin Reverse | GCCGGACTCATCGTACTCC |
